# Diagnostic accuracy of the Finnish Diabetes Risk Score for the prediction of undiagnosed type 2 diabetes, prediabetes, and metabolic syndrome in the Lebanese University

**DOI:** 10.1186/s13098-020-00590-8

**Published:** 2020-09-30

**Authors:** Maher Abdallah, Safa Sharbaji, Marwa Sharbaji, Zeina Daher, Tarek Faour, Zeinab Mansour, Mohammad Hneino

**Affiliations:** 1 Faculty of Public Health, Lebanese University, Hadat, Beirut, Lebanon; 2 Department of Nutrition and Dietetics, Faculty of Public Health, Lebanese University, Hadat, Beirut, Lebanon; 3 Medical Laboratory, Lebanese University Medical Center, Lebanese University, Hadat, Beirut, Lebanon; 4 Sciences Department, Faculty of Public Health, Lebanese University Hadat, Hadat, Beirut, Lebanon

**Keywords:** AUROC, Cutoff, FBG, FINDRISC, MS, OGTT, Prediabetes, Screening, UT2DM, WC

## Abstract

**Background:**

Risk scores were mainly proved to predict undiagnosed type 2 diabetes mellitus (UT2DM) in a non-invasive manner and to guide earlier clinical treatment. The objective of the present study was to assess the performance of the Finnish Diabetes Risk Score (FINDRISC) for detecting three outcomes: UT2DM, prediabetes, and the metabolic syndrome (MS).

**Methods:**

This was a prospective, cross-sectional study during which employees aged between 30 and 64, with no known diabetes and working within the faculties of the Lebanese University (LU) were conveniently recruited. Participants completed the FINDRISC questionnaire and their glucose levels were examined using both fasting blood glucose (FBG) and oral glucose tolerance tests (OGTT). Furthermore, they underwent lipid profile tests with anthropometry.

**Results:**

Of 713 subjects, 397 subjects (55.2% female; 44.8% male) completed the blood tests and thus were considered as the sample population. 7.6% had UT2DM, 22.9% prediabetes and 35.8% had MS, where men had higher prevalence than women for these 3 outcomes (*P* = 0.001, *P* = 0.003 and *P* = 0.001) respectively. The AUROC value with 95% Confidence Interval (CI) for detecting UT2DM was 0.795 (0.822 in men and 0.725 in women), 0.621(0.648 in men and 0.59 in women) for prediabetes and 0.710 (0.734 in men and 0.705 in women) for MS. The correspondent optimal cut-off point for UT2DM was 11.5 (sensitivity = 83.3% and specificity = 61.3%), 9.5 for prediabetes (sensitivity = 73.6% and specificity = 43.1%) and 10.5 (sensitivity = 69.7%; specificity = 56.5%) for MS.

**Conclusion:**

The FINDRISC can be considered a simple, quick, inexpensive, and non-invasive instrument to use in a Lebanese community of working people who are unaware of their health status and who usually report being extremely busy because of their daily hectic work for the screening of UT2DM and MS. However, it poorly screens for prediabetes in this context.

## Background

Diabetes mellitus (DM) is a chronic metabolic disorder characterized by persistent hyperglycemia [1]. It is preceded by an asymptomatic state known as prediabetes which might begin 12 years before the diagnosis, although glucose values seemed to be tightly regulated within the normal range until 2–6 years before the clinical diagnosis when a sharp increase was detected [2]. In fact, during this stage insulin resistance occurs and triggers damage to several organs, such as eyes, kidneys, blood vessels, and the heart [3]. Furthermore, insulin resistance is associated with hyperinsulinemia which is the underlying cause of the MS and, once acquired, those with a genetic predisposition would develop the other features of the disorder including hypertension, hypertriglyceridemia, low high-density lipoprotein cholesterol (HDL-C), and the development of type 2 diabetes (T2D) [4].

The prevalence of diabetes, prediabetes, and MS is increasing rapidly worldwide [5, 6]. According to the international diabetes federation (IDF), approximately 38.7 million people or 9.6% of adults aged 20–79 years suffer from diabetes which will likely double in 2045 and about 49.1% of these are undiagnosed in the IDF—the Middle East North Africa region (MENA) [7]. In Lebanon, the prevalence of diabetes was 14% in 2017 compared to 12.6% according to the World Health Organization (WHO) in 2016 [8]. Similarly, the prevalence of diabetes and prediabetes was found to be 15% and 40.3% respectively in the Greater Beirut Area in 2017 [9]. Retinopathy, heart disease, and neuropathy have been the most highly correlated complications with diabetes among the Lebanese population and impose a serious economic burden on the healthcare system [10, 11]. Moreover, the prevalence of MS in the Middle East countries was estimated to be 25% in 2017[12]. Interestingly, Prediabetes and diabetes were both positively correlated with the different components of the MS within the Lebanese population [11].

Given all these pieces of evidence, early detection and treatment of prediabetes, diabetes, and MS would delay progression to diabetes as well as its relevant health and economic burdens [3, 7, 13]. Indeed, a two-step strategy could be efficient and highly recommended by many guidelines [14, 15]. First, rapid preliminary screening is performed to identify high-risk individuals using risk assessment tools. Secondly, they must be referred for routine blood measures for a final definitive diagnosis.

Till now, several risk assessment tools have been developed for initial screening. However, many of them are not widely accepted and practically used since they may require invasive and expensive blood testing such as the Atherosclerosis Risk in Communities score (ARIC) and the Framingham Offspring score [2, 15]. In contrast, there are also non-invasive risk scores that rely only on self-reported data. The well-known ones are the ADA risk score [16], the German Diabetes Risk Score (GDRS) [17], and the Finnish Diabetes Risk Score (FINDRISC). The latter has been most frequently tested for detecting diabetes risk [7, 18]. However, few studies examined its ability to predict MS. Besides, as an external validation must be done in the population in which they are intended to be used [15, 19] we aimed to evaluate the performance of the FINDRISC in detecting UT2DM, prediabetes, and MS in a Lebanese community.

## Methods

### Study design and participants

This was a prospective, cross-sectional study, conducted between January 2018 and May 2019 over two phases. During the first phase, the staffs working within the faculties of the LU (Office workers, instructors, and cleansers), aged older than 30 to 64 years and who accept to submit the inform consent were recruited by convenience sampling from the campuses of the LU across four regions in Lebanon (Beirut, Bekaa, South, and North) and filled the FINDRISC questionnaire. However, individuals who reported having diabetes, women who reported to be pregnant, and people with any physical disability that prevents anthropometric measurements were excluded. In the second phase, faculties were contacted in advance by phone for a blood test analysis appointment.

### Measurements

#### Questionnaire

FINDRISC was originally used as a prediction tool to identify patients at risk of developing diabetes over next 10 years [20]. It consists of eight, self-reported questions related to age, Body mass index (BMI), physical activity, vegetable and fruit intake, medical treatment of hypertension, history of hyperglycemia and, diabetes family history. A rating score between 0 and 14 points indicates a low to moderate risk of diabetes; a risk score between 15 and 20 points indicates a high risk of diabetes and a rating score of more than 20 points indicates a very high risk of diabetes. After signing the informed consent, all participants completed the questionnaire that included the original eight items of FINDRISC, with additional information addressing the socio-demographic data, smoking, and educational level.

#### Anthropometrics

Bodyweight and waist circumference (WC) were measured for each participant by Nutrition students [21]. Weight was measured to the nearest 0.1 kg on a calibrated digital scale, with light clothing and without shoes. Using a flexible measuring tape, WC was measured midway between the lowest rib and the iliac crest. However, the height was self-reported. Then, BMI was calculated by dividing body mass in kilograms by height in meters squared [22].

#### Resting Blood Pressure

Blood Pressure measurement was taken after the participant had been seated and relaxed for 5 min without any distractions, using an automatic monitor (Ross max monitoring, Swiss design) with appropriate cuff’s size. Furthermore, the person’s upper arm was put into the cuff loops, 1 or 2 cm above the elbow, then letting it comfortably rest on the table [23]. Those who had a blood pressure level higher than 120/80 mmHg upon measurement, were notified to seek medical advice.

#### Laboratory measurements

Participants were instructed to fast for at least 12 h and abstain from vigorous exercise in the evening and the morning of the investigation. They were also asked to abstain from caffeine and smoking on the morning of the visit. After ensuring that the fasting period was accomplished completely, the blood sampling procedure was explained by a trained laboratory technician. A total of 3–5 ml of fasting venous blood sample was collected in a serum clot activator tube and centrifuged at 4000 rpm for 10 min on the same day, and then serum was transferred to another tube and stored at -22 °C for biochemical examination. Following this, a load of 75 g of anhydrous glucose in a volume of 200 ml was administered to each individual for the oral glucose tolerance test (OGTT) [14]. After two hours, a second blood sample was drawn to assess the glucose levels. Fasting blood glucose (FBG), triglycerides (TG), total cholesterol (TC), and high-density lipoprotein-cholesterol (HDL-C) levels were detected using a biochemical analyzer (Unicel DxC 600, Synchron Clinical System, BECKMAN COULTER, Cobas C111). However, low-density lipoprotein-cholesterol (LDL-C) level was calculated by the Freidwald formula (24), only if the total TG level did not exceed 300 mg/dl. All these blood analysis procedures were conducted in certified laboratories located in each region (Lebanese University Medical Centre, Mount Lebanon; Hammoud hospital, South Lebanon; Libano Français Hospital, Bekaa and Tripoli Medical Center, North Lebanon). The results of the blood tests were provided to the participants.

### Outcomes

We had three outcome variables of interest: UT2DM, Prediabetes, and MS.

Both UT2DM and Prediabetes were defined according to the latest American Diabetes Association (ADA) criteria [14].

- Individuals who had FBG level ≥ 126 mg/dl (7.0 mmol/l) or 2-h blood glucose (2-h BG) ≥ 200 mg/dl (11.1 mmol/l) were classified as UT2DM.
- Participants who had impaired fasting glucose (IFG) i.e. blood glucose (BG) ≥ 100 (5.6 mmol/l) and < 126 mg/dl (7.0 mmol/l) or impaired glucose tolerance (IGT) i.e. 2 h blood glucose (2 h BG) ≥ 140 (7.8 mmol/l) and < 200 mg/dl (11.1 mmol/l) were considered as prediabetics.

Whereas, MS was defined according to the latest National Cholesterol Education Program Adult treatment panel III (NCEP ATP III) diagnostic criteria (2005revision) [25]. At least three of the following criteria were present:

- Abdominal obesity (WC ≥ 102 cm in men and ≥ 88 cm in women)
- Hypertriglyceridemia (TG ≥ 150 mg/dl or 1.695 mmol/l)
- Low HDL-C (HDL < 40 mg/dl in men and < 50 mg/dl in women)
- Elevated blood pressure (Systolic blood pressure SBP > 130 mmHg or Diastolic blood pressure DBP > 85 mmHg) or the use of antihypertensive medication.
- Elevated blood glucose (FBG ≥ 100 mg/dl).

### Statistical analyses

Statistical analysis was conducted using SPSS (IBM Corp, SPSS Statistics version 23). Descriptive statistics of those who underwent blood tests were expressed as means (± standard deviation) for continuous variables and as proportions for categorical variables. Differences in the socio-demographic (SD) variables between genders were computed using an independent samples t-test for continuous variables and a Chi-square test for categorical variables, while between FINDRISC categories using a one-way ANOVA test for continuous variables and a Chi-square test for categorical variables. To evaluate the FINDRISC accuracy performance we calculated the area under the receiver operating curve (AUROC), sensitivity (the probability that the test is positive for subjects with type 2 diabetes), specificity (the probability that the test is negative for subjects without type 2 diabetes) with 95% CIs (95% confidence intervals). To create the ROC curve, sensitivity was plotted on the *y-axis*, and the false-positive rate (1-specificity) was plotted on the *x*-axis. Then optimal cut-off points were determined by the point with the closest distance to (0; 1) in the ROC curve which maximizes the sensitivity and specificity of the test (Tradeoff between sensitivity and specificity).

## Results

### Participant characteristics

Out of 713 individuals who were initially enrolled in the study and filled the questionnaire, 316 (44.3%) did not follow the complete blood tests procedures; and therefore, the data of the remaining 397 were used for the validation of the FINDRISC. The sample was composed of 219 women (55.2%) and 178 men (44.8%). The mean age was 48.5 (± 9) and there was no statistical significance between men and women in terms of age (*P* = 0.248). Men had higher BMI (28.5 vs. 26.3; *P* < 0.001), WC (102.8 vs. 90.5; *P* < 0.001), were heavier smokers (172 vs. 137; *P* < 0.001) and were on BP medication more than women (35 vs. 28; *P* = 0.042). However, women had a stronger 1^st^ degree family history of T2DM (92 vs.76; *P* = 0.002) and were relatively less physically active (21 vs. 38; *P* = 0.001). The mean values of the blood tests including FBG (*P* < 0.001), OGTT (P < 0.001), TG (*P* < 0.001), SBP (*P* = 0.001) and DBP (*P* = 0.003) were higher in men than in women, except for HDL-C (*P* < 0.001), LDL-C (*P* = 0.006) and TC (*P* < 0.001) (Table 1).

**Table 1:** FINDRISC components and socio-demographic characteristics according to gender

	Overall (N = 397)	Gender		*P*
		Men	Women	
		(n = 178, 44.8%)	(n = 219, 55.2%)	
FINDRISC components				
Age (in years)	48.4 (± 9)	49.06 (± 9.6)	48 (± 8.4)	0.248
Family Hx for T2DM				
No family history	169 (42.6%)	87 (48.9%)	82 (37.4%)	0.002*
1^st^ degree relatives	168 (42.3%)	76 (42.7%)	92 (42%)	
2^nd^ degree relatives	60 (15.1%)	15 (8.4%)	45 (20.5%)	
WC (in cm)	96 (± 14.3)	102.8 (± 12.1)	90.5 (± 13.6)	0.000*
PA ≥ 30 in min/day	59 (14.9%)	38 (21.3%)	21 (9.6%)	0.001*
Daily intake of F&V	227 (57.3%)	95 (53.4%)	132 (60.6%)	0.091
Use of BP medication	63 (15.9%)	35 (19.7%)	28 (12.8%)	0.042*
Hx of high BG	42 (10.6%)	24 (13.5%)	18 (8.2%)	0.063
BMI (in kg/m^2^)	27.3 (± 4.7)	28.5 (± 4.5)	26.3 (± 4.6)	0.000*
Socio-demographic characteristics				
Smoking				0.000*
Never	225 (56.7%)	84 (47.2%)	141 (64.4%)	
Former	17 (4.3%)	15 (8.4%)	2 (0.9%)	
Current	309 (43.3%)	172 (49.3%)	137 (37.6%)	
Educational level (as degree)				
Less than brevet	48 (12.1%)	28 (15.7%)	20 (9.1%)	0.105
Brevet	19 (4.8%)	11 (6.2%)	8 (3.6%)	
Baccalaureate	70 (17.6%)	28 (15.7%)	42 (19.2%)	
Bachelor	96 (24.2%)	36 (20.2%)	60 (27.4%)	
Master’s and above	164 (41.3%)	75 (42.1%)	89 (40.6%)	
Family income (in LBP)				
< 1 million	38 (9.6%)	15 (8.4%)	23 (10.5%)	0.000*
1 million-2 million	74 (18.6%)	43 (24.2%)	31 (14.2%)	
2 million-3 million	114 (28.7%)	34 (19.1%)	80 (36.5%)	
> 3 million	171 (43.1%)	86 (48.3%)	85 (38.8%)	

*Statistically significance

Data are presented as means (±SD) for continuous variables and as frequencies (percentage) for categorical variables. *Hx* history, *T2DM* type 2 diabetes mellitus, *WC* waist circumference, *PA* physical activity, *F&V* fruits and vegetables, *BP* blood pressure, *BG* blood glucose, *BMI* body mass index, *LBP* Lebanese pound

### Prevalence of UT2DM, prediabetes, and MS

30 (7.6%) individuals had UT2DM, 91 (22.9%) had prediabetes and 142 (35.8%) had MS. In addition, Men had statistically higher prevalence of UT2DM (*P* = 0.001), prediabetes (*P* = 0.003) and MS (*P* = 0.001) (Table 2). No clinical side effects were reported following the procedure of blood sampling.

**Table 2:** Blood tests and the three outcome variables according to gender

	Overall (N = 397)	Gender		*P*
		Men (n = 178, 44.8%)	Women (n = 219, 55.2%)	
Blood tests				
FBG (in mg/dl)	96 (± 30.3)	102.70 (± 41.639)	91.53 (± 14.325)	0.000*
2 h OGTT (in mg/dl)	111 (± 64.8)	125.74 (± 85.688)	99.70 (± 36.985)	0.000*
TC (in mg/dl)	198 (± 41.2)	189.71 (± 42.258)	205.20 (± 39.196)	0.000*
TG (in mg/dl)	139 (± 87.8)	160.97 (± 98.770)	122.65 (± 73.715)	0.000*
HDL-C (in mg/dl)	48 (± 13.6)	41.56 (± 11.083)	54.04 (± 12.954)	0.000 *
LDL-C (in mg/dl)	123 (± 36.9)	117.39 (± 39.656)	127.71 (± 33.955)	0.006*
SBP (in mmHg)	121 (± 23.4)	125.48 (± 25.957)	117.65 (± 20.610)	0.001*
DBP (in mmHg)	77 (± 15.3)	80.29 (± 17.013)	75.49 (± 13.547)	0.003*
FINDRISC score	10 (± 4.2)	11.07 (± 4.685)	10.79 (± 3.958)	0.528
Outcomes				
UT2DM	30 (7.6%)	22 (12.4%)	8 (3.7%)	0.001*
Prediabetes	91 (22.9%)	53 (29.8%)	38 (17.4%)	0.003*
MS	142 (35.8%)	85 (47.7%)	57 (26%)	0.000*

*Statistical significance

*FBG* fasting blood glucose, *2h OGTT* 2 hours oral glucose tolerance test, *TC* total cholesterol, *TG* triglyceride, *HDL-C* high density lipoprotein cholesterol, *LDL-C* low density lipoprotein cholesterol, *SBP* systolic blood pressure, *DBP* diastolic blood pressure, *UTDM2* undiagnosed type 2 diabetes mellitus, *MS* metabolic syndrome

## Diagnostic accuracy of FINDRISC in detecting UT2DM, prediabetes and MS

### Diagnostic accuracy for UT2DM

**Fig. 1:**
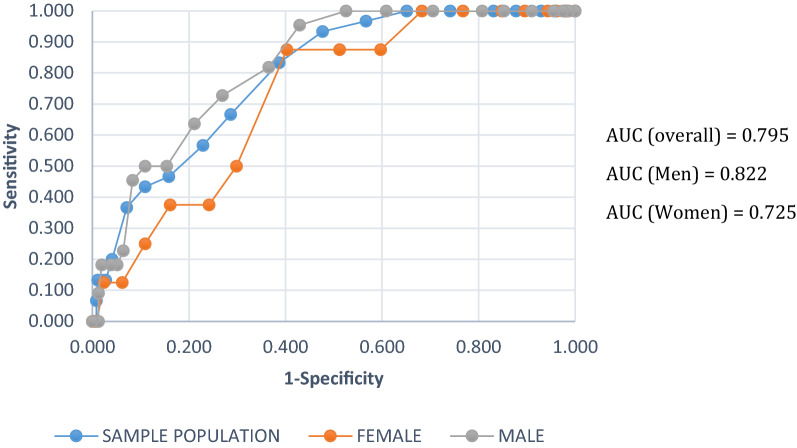
ROC curve for UT2DM for the sample population and by gender

The AUROC curve for detecting UT2DM was 0.795 (95% CI: 0.728–0.862) overall, with 0.822 (0.749–0.895) for men better than, 0.725 (0.589–0.861) for women (Fig. 1). The correspondent optimal cutoff point was a FINDRISC equal to 11.5, at which the sensitivity was 83.3% and specificity was 61.3% (Table 3). Similarly, the optimal cut-off value for women was 11.5 (sensitivity 87.5%; specificity 60%) but 10.5 for men (sensitivity 95.5%; specificity 57.1%).

**Table 3:** FINDRISC threshold values for detecting UT2DM, prediabetes and MS according to sensitivity and specificity

Cutoff points	Sensitivity (%)	Specificity (%)
UT2DM		
Cuttoff = 9.5	96.70	43.30
Cuttoff = 10.5	93.30	52.30
* Cuttoff = 11.5*	*83.30*	*61.30*
Prediabetes		
* Cuttoff = 9.5*	*74.70*	*43.10*
Cuttoff = 10.5	61.50	49.70
Cuttoff = 11.5	57.10	61.10
MS		
Cuttoff = 9.5	77.50	48.20
* Cuttoff = 10.5*	*69.70*	*56.50*
Cuttoff = 11.5	62	67.50

### Diagnostic accuracy for prediabetes

**Fig. 2:**
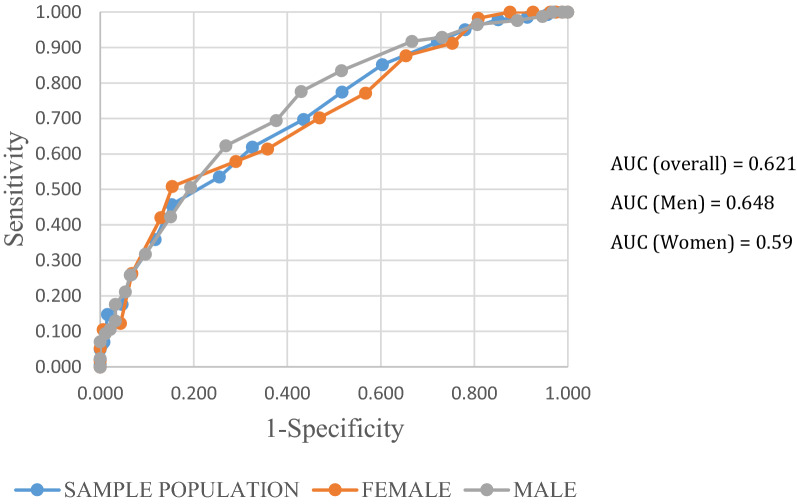
ROC curve for prediabetes for the sample population and by gender

The AUROC curve for detecting prediabetes was 0.621 (95% CI: 0.557–0.684) overall, with 0.648 (0.563–0.734) for men better than, 0.59 ~ 0.6 (0.492–0.687) for women (Fig. 2). The correspondent optimal cutoff point was a FINDRISC equal to 9.5, at which the sensitivity was 73.6% and specificity was 43.1% (Table 3). Similarly, the optimal cut-off value for both men and women was 9.5 with (sensitivity 77.4%; specificity 48%) for men and (sensitivity 71.1%; specificity 39.8%) for women.

### Diagnostic accuracy for MS

**Fig. 3:**
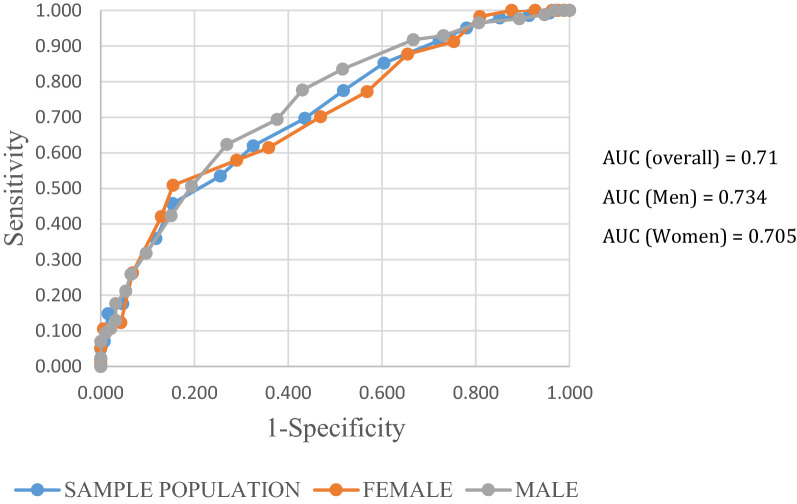
ROC curve for MS for the sample population and by gender

The AUROC curve for detecting MS was 0.71 (95% CI: 0.657–0.762) overall, with 0.734 (0.661–0.807) for men slightly better than, 0.705 (95% CI: 0.626–0.784) for women (Fig. 3). The correspondent optimal cutoff point was a FINDRISC equal to 10.5, at which the sensitivity was 69.7% and specificity was 56.5% (Table 3). Similarly, the optimal cut-off value for men was 9.5 (sensitivity 77.6%; specificity 57%) while it was 10.5 for women (sensitivity 70.2%; specificity 53.1%).

### Characteristics of the participants according to FINDRISC categories

The highest prevalence of most participants characteristics fall in the highest FINDRISC category > 20, with a remarkable statistical significance, which are WC 112.5 (± 10.4; *P* < 0.001), BMI 32 (± 5.2; *P* < 0.001), FBG 110.2 (± 27; P = 0.036), OGTT 170.6 (± 79.8; P < 0.001), TG 227 (± 154.2; P = 0.003), HDL-C 35.2 (± 7.1, P = 0.005), SBP 138.2 (± 13.2; P < 0.001) and, DBP 88.2 (± 14.8; P = 0.038). Subjects with a FINDRISC category of 15–20 had the highest prevalence of history of elevated blood glucose 22 (52.4%; P < 0.001), UT2DM 11 (36.7%; P < 0.001) and, TC 14 (46.7%; *P* < 0.001 = 0.645). Older individuals 51.4 (± 8.6; P < 0.001), current smokers 35 (22.6%; P = 0.522) and, those with elevated blood LDL-C levels 126.8 (± 38.1; P = 0.247) are located within a FINDRISC category of 12–14, with a statistical significant except for smokers. Those with prediabetes 33 (36.3%; P = 0.01) and MS 51 (34.5%; P < 0.001) had a FINDRISC category of 7–11 (Table 4).

**Table 4:** Characteristics of the participants according to FINDRISC categories

	FINDRISC categories					*P*
	< 7	7–11	12–14	15–20	> 20	
Age (in years)	42.9 (± 9.08)	47.6 (± 8.2)	*51.4* (± 8.6)	51.3 (± 8.3)	50.1 (± 10.2)	0.000*
WC (in cm)	84.2 (± 11.3)	94.2 (± 12.2)	98.8 (± 12.61)	105 (± 14.7)	*112.5* (± 10.4)	0.000*
BMI (in kg/m^2^)	23.4 (± 2.36)	26.4 (± 3.8)	28.3 (± 4.6)	30.7 (± 4.87)	*32.5* (± 5.2)	0.000*
Hx of high BG	0 (0.0%)	3 (7.1%)	7 (16.7%)	*22* (52.4%)	10 (23.8%)	0.000*
Current smoking	26 (16.8%)	59 (38.1%)	*35* (22.6%)	28 (18.1%)	7 (4.5%)	0.522
UT2DM	0 (0.0%)	6 (20.0%)	9 (30.0%)	*11* (36.7%)	4 (13.3%)	0.000*
Prediabetes	6 (6.6%)	*33* (36.3%)	27 (29.7%)	23 (25.3%)	2 (2.2%)	0.01*
MS	8 (5.4%)	*51* (34.5%)	38 (25.7%)	42 (28.4%)	9 (6.1%)	0.000*
FBG (in mg/dl)	88.6 (± 8.3)	95 (± 35.7)	97.4 (± 22)	103 (± 36.4)	*110.2* (± 27)	0.036*
2 h OGTT (in mg/dl)	89.1 (± 23.5)	105 (± 63.6)	117.6 (± 59.7)	128 (± 84.6)	*170.6* (± 79.8)	0.000*
TC (in mg/dl)	197 (± 42.1)	197 (± 40.3)	198.9 (± 43)	*201* (± 41.3)	180.2 (± 35.2)	0.635
TG (in mg/dl)	121 (± 62.9)	133 (± 76)	146 (± 105.5)	151 (± 88.6)	*227* (± 154.2)	0.003*
HDL-C (in mg/dl)	51.9 (± 14.6)	48 (± 12.5)	46.7 (± 14)	48 (± 14.2)	*35.2* (± 7.1)	0.005*
LDL-C (in mg/dl)	120 (± 37.4)	122 (± 35.7)	*126.8* (± 38.1)	126 (± 37.6)	99.7 (± 33.5)	0.247*
SBP (in mmHg)	114 (± 19.3)	118 (± 24.8)	121.2 (± 23.6)	130 (± 20.6)	*138.2* (± 13.2)	0.000*
DBP (in mmHg)	75 (± 12.9)	76 (± 16.3)	78.6 (± 15)	80 (± 14.3)	*88.2* (± 14.8)	0.038*

*Statistical significance

## Discussion

### Key findings

In this cross-sectional study, FINDRISC had a good performance in identifying UT2DM and MS in the working population (Office workers, instructors, and cleaners) of the LU Campuses, but a poor performance regarding prediabetes.

### Comparison with other studies

#### Prevalence of UT2DM, prediabetes, and MS

The prevalence of UT2DM and prediabetes in our study was 7.6% and 22.9% respectively. However, a recent study carried out in the Bekaa, which is a rural area in Lebanon [26] found that the estimates of diabetes and prediabetes were 26% and 8.5% respectively using a sample of 200 individuals. Nevertheless, the prevalence of diabetes and prediabetes was reported to be 15% and 40.3% respectively in the Greater Beirut Area in a sample of 501 people [9]. These findings indicate that the prevalence of both diabetes and prediabetes are high in different Lebanese settings. As for MS prevalence, it was estimated to be 36% among LU employees. Similarly, a recent cross-sectional study has been carried out in Notre Dame University employees on the three campuses (Zouk Mosbeh, North and Al Chouf) and found that 23.5% of the participants were suffering from MS [27]. These findings are alarming, suggesting that LU employees are, in general, unaware of their health status which is highlighted by a low percent of physical activity practice (85%) [28], high waist circumference especially for men (102.8 ± 12.1 cm) [29] and an overweight population [30]. These factors have been largely discussed and identified as risk factors for diabetes and 'metabolic syndrome' and associated health problems. Thus, the importance of the FINDRISC use among them is highlighted.

#### Performance of FINDRISC in detecting UT2DM, prediabetes and MS

Originally, the FINDRISC questionnaire was developed longitudinally as a future predictor of diabetes in the Finnish population [20] and was validated from a multivariate logistic regression model five years later. It was subsequently cross-sectionally validated using a maximum score of 26 [31]. Later on, it has been assessed in a cross-sectional manner in several Asian [32–34], European [35–42], and American countries [43–45]. In these studies, the optimal cut-off points for detecting UT2DM varied widely from 8.5 to 17 with a sensitivity ranging from 48 to 84% and a specificity ranging from 30.9% to 95%. Also, the AUROC went from 0.569 to 0.88. This vast variability indicates the need for assessing the tool within its target population.

In this study, FINDRISC had a good discriminative ability for detecting UT2DM with an AUROC value of 0.795 (0.822 in men; 0.725 in women) and a threshold value of 11.5 (10.5 in men; 11.5 in women). Besides, it’s also good at detecting MS in both men and women with an AUROC of 0.7 (0.713 in men; 0.708 in women) at a threshold of 10.5 (9.5 in men; 10.5 in women). Whereas, this ability gets weaker in case of prediabetes as evidenced by an AUROC of 0.621 (0.648 in men; 0.59 in women) especially in women. Several studies showed similar values and also confirmed that FINDRISC performed better in detecting UT2DM and MS than prediabetes. In a previous cross-sectional study on the general population of the United States of America [45], the AUROC for UT2DM was 0.75 (0.74 in men; 0.78 in women) with an optimal cutoff value of 11 in men and 12 in women, while the AUROC for prediabetes was 0.67 (0.66 in men; 0.7 in women) with an optimal cutoff of 9 in men and 10 in women. Likewise, in the Philippines [32], FINDRISC was good at predicting T2DM with an AUROC of 0.738 (0.749 in men; 0.734 in women) but failed to screen for prediabetes (AUROC = 0.562). Similar trends were noticed in the original FINDRISC study regarding the performance of the tool in detecting the three outcomes [31]. In other words, the AUROC for MS discrimination was 0.72 in men and 0.75 in women. However, the optimal cutoff values for detecting T2DM and prediabetes were both 11 with lower sensitivities and specificities than the ones found in this study, and the optimum cutoff for MS was not established. Similarly, FINDRISC was also found to perform well in the detection of MS (AUC = 0.77) in Taiwanese [46], but the optimal cutoff point was not reported. One previous cross-sectional study in Greece [35] reported a threshold for MS of 15 which is higher than the one reported in our study. However, it is well known that prediabetes which is a combination of excess body fat and insulin resistance, is considered an underlying etiology of MS [47]. In turns, MS is considered as a risk factor for T2DM [48] which may explain why 70% of people with prediabetes in this study had MS (*P* < 0.0001) and 76% of those with UT2DM had MS and that’s why the threshold for MS is localized between the thresholds for prediabetes and UT2DM in our community. To date, only one study assessed the predictive ability of FINDRISC in detecting incident cases of MS (AUC = 0.65) rather than prevalent cases at a cutoff of 12 [49].

It is also worth mentioning that men had always higher AUROC values as well as lower cutoff values than women, specifically for UT2DM and MS in the current study. In other words, men tend to have more risk factors putting them at a higher risk for diabetes, prediabetes, and MS which improves the predictive ability of FINDRISC when compared with women and increases their scoring in FINDRISC and thus limiting their threshold to lower values. In this study, a synergistic interaction for the combined BMI (p < 0.0001), WC (p < 0.0001), smoking (p < 0.0001), could renders men more prone for diabetes with higher prevalence for UT2DM (p = 0.001) and MS (p < 0.0001).

#### The usefulness of FINDRISC as a screening tool among LU workers

The advantage of the FINDRISC relies on its self-report questions so that LU workers that reported to be extremely busy because of their work and daily life stressors can find it easier to fill the FINDRISC quickly and rate their current health status. Being at higher risk based on FINDRIC score would be a sufficient trigger for them to start applying lifestyle changes or to seek health professionals’ help.

### Strengths and limitations of the study

Some limitations warrant considerations. First, a misclassification bias could be introduced because the diagnosis of diabetes of the respondents was self-reported. Further, the diagnosis of diabetes and prediabetes of the included participants was not confirmed by repeat testing on a separate day as recommended [14]. However, these tests may pose additional costs on our limited budget. Second, a selection bias could be present as the participants were drawn only from LU campuses and, thus, the results may not be generalizable to the rest of the Lebanese citizens living in other settings. Third, we could not assess the ability of the FINDRISC to catch the future risk of having diabetes and MS as it was tested in some longitudinal studies [49–51].

This study has also considerable strengths. To our knowledge, this is the second study that has been carried out in an Arabic country in the Middle East region which has investigated the validity of FINDRISC. A previous study was conducted in Kuwait and showed similar results [33]. Additionally, a recent Jordanian study pointed out the usefulness of FINDRISC to screen for type 2 diabetes in a young student population but didn’t have the opportunity to validate it [52]. Second, a selection bias was avoided since our sample was fairly divided between men (44.8%) and women (50.2%) and thus the gender differences in the study outputs are not biased. Third, the diagnosis of diabetes was done based on a combination of two plasmatic tests as it is ideally recommended which are the FBG and OGTT. Thus, the misclassification bias would be lessened, and the estimation of the risk of T2DM as well as the performance of FINDRISC are optimized.

## Conclusion and perspectives

This cross-sectional study has successfully demonstrated that FINDRISC could be useful as a first-line screening tool that identifies employees with UT2DM, prediabetes, and MS that might benefit from lifestyle modification. FINDRISC model could be also beneficial for community-based interventions and screenings as well as in clinical practice by the health professionals. In future studies, FINDRISC should be validated on a larger and more representative sample of the Lebanese population so Lebanese citizens living in a resource-poor setting like rural areas would benefit the most. Also, FINDRISC should be assessed in a longitudinal study who allows the identification of incident cases of diabetes and MS rather than prevalent cases.

## Data Availability

Not applicable.
